# Spatial pattern of tuberculosis (TB) and related socio-environmental factors in South Korea, 2008-2016

**DOI:** 10.1371/journal.pone.0255727

**Published:** 2021-08-05

**Authors:** Changmin Im, Youngho Kim

**Affiliations:** 1 Department of Geography, Graduate School, Korea University, Seoul, South Korea; 2 Department of Geography & Geography Education, Korea University, Seoul, South Korea; The University of the South Pacific, FIJI

## Abstract

Tuberculosis (TB) incidence and corresponding mortality rates in S. Korea are unusual and unique compared to other economically developed countries. Korea has the highest TB incidence rate in Organization for Economic Co-operation and Development (OECD) countries. TB is known as a disease reflecting socio-economic and environmental conditions of a society. Besides, TB is an infectious disease spread through the air, naturally forming spatial dependence of its incidence. This study investigates TB incidences in Korea in socio-economic and environmental perspectives. Eigenvector spatial filtering applied accounts for spatial autocorrelation in the TB incidence, and Getis-Ord $G_{i}^{*}$ statistic tracks the changes of TB clusters at given time. The results show that population composition ratio, population growth rate, health insurance payment, and public health variables are significant throughout the study period. Environmental variables make minor effects on TB incidence. This study argues that unique demographic features of Korea are a potential threat to TB control in the future.

## Introduction

Tuberculosis (TB) incidence and corresponding mortalities are at a dangerous level in South Korea. TB incidence rate in Korea is the highest in Organization for Economic Cooperation and Development (OECD) countries [1]. Most OECD members are regarded as developed countries with high-income economies. Seeing that TB incidences are frequently observed in economically underdeveloped countries [2], TB occurrences reflect the socio-economic status of the countries [3–5]. Korea is categorized as one of the highly developed countries, being ranked 12th in GDP [6] and 15th in national competitiveness in 2018 [7]. Particularly in healthcare resources, Korea is in the world’s top-class [8]. Consequently, given her social and economic development, it is only natural to expect very low TB incidence rate in Korea.

People’s expectation of TB incidence is quite different from actual occurrences in Korea. Although 32.2% adult Korean believe that TB has totally been eradicated [9], actual TB incidence has been prevalent. Furthermore, MDR (Multidrug-resistant) TB incidence and recurrence risk are one of the highest in the world [10]. South Korea also has a rapidly aging population. From 2001 to 2016, TB deaths for those aged 80 and over has increased by about 35% [11].

This unique situation of Korean TB incidence has drawn attention from international research institutes such as World Health Organization (WHO) [12]. Including Korea Centers for Disease Control & Prevention (KCDC), almost all Korean TB studies have approached the disease through biomedical perspectives, ignoring socio-environmental factors [13]. As a result, specific mechanisms for TB incidence have yet to be identified [14].

TB is a social disease. Socio-environmental factors affect TB incidence. Recent TB studies consider both biomedical and socio-environmental perspectives together [15,16]. Observing the global trend in the past 200 years [4], socio-environmental factors such as population, economic status, and outdoor air pollution have influenced TB incidence in addition to biological factors. Many contemporary TB studies show great interest in socio-environmental factors [3,5,17].

A TB analysis must account for spatial aspects. TB is a contagious disease and transmitted through air [18–20]. Naturally, TB incidence data are not independent, but similar values are located nearby, leading to a correlation among data [21,22]. TB incidence data contain spatial dependence [23–26]. However, many TB studies ignored spatial perspectives of the incidence [10,27,28], which lead to biased or inefficient analysis results in socio-environmental analysis [29,30].

This study is distinguished by considering spatial perspectives of TB incidence in Korea. This study analyzes TB incidence using eigenvector spatial filtering model (ESFM) and accounts for spatial perspective of the disease. The results show that TB incidences are significantly influenced by socio-economic and environmental variables such as population composition ratio, population growth rate, health insurance payment, and public health throughout the study period from 2008 to 2016. Getis-Ord $G_{i}^{*}$ statistics [29,31] and corresponding cluster analysis present hotspots in the eastern parts of Korea.

## Materials and methods

### Study scope and data

This study covers all territory of Korea, composed of 245 Si, Gun, and Gu administrative areal units (city and county level units). Minor islands were excluded because of their negligible population size and separate locations from the mainland. TB incidences from 2008 to 2016 were analyzed. TB incidence data were acquired from the annual reports of the Korea Centers for Disease Control and Prevention (KCDC, https://www.cdc.go.kr).

**Fig 1:**
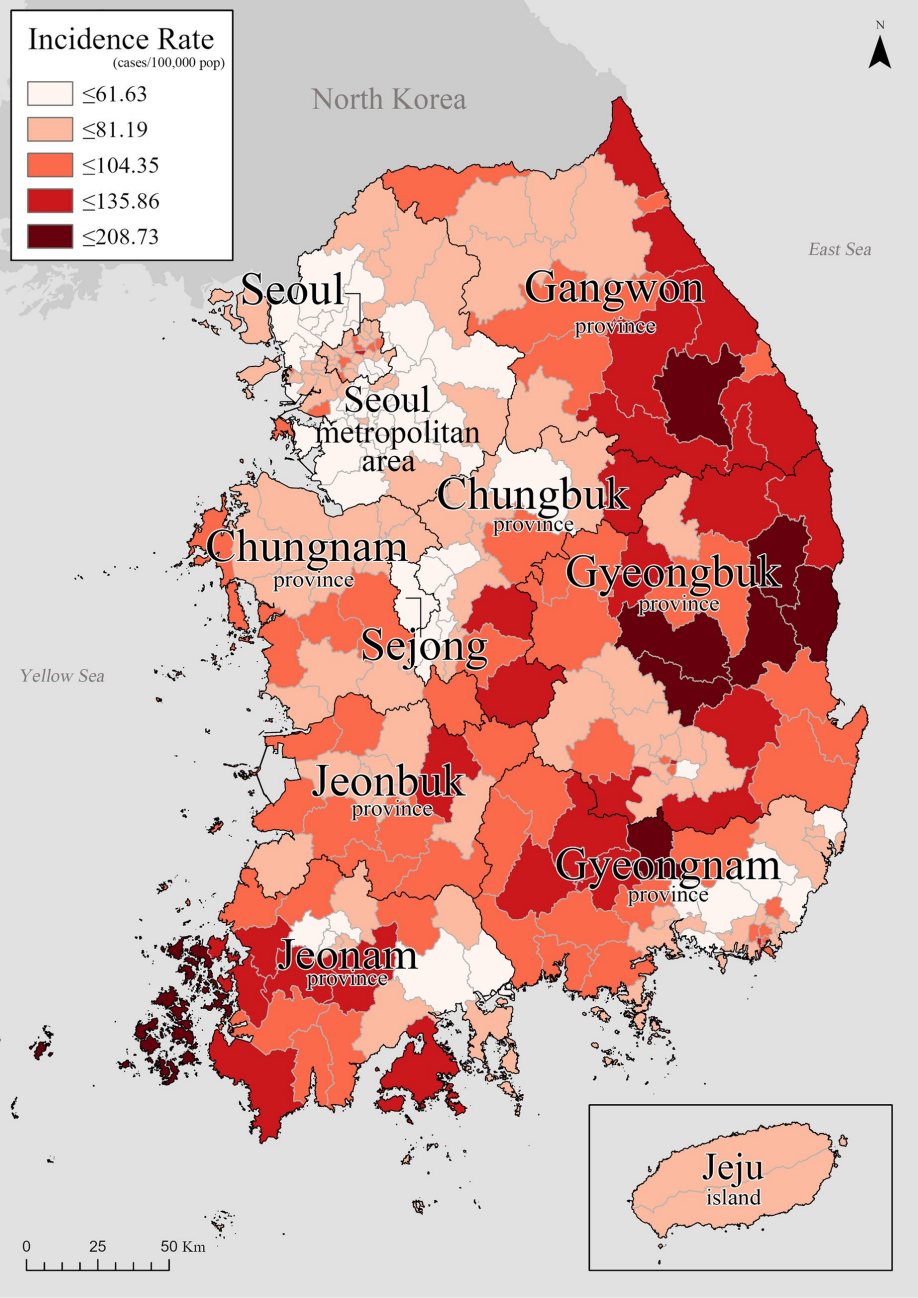
Study area and TB incidence rate (cases/100,000).

Fig 1 shows the average incidence rate of TB in Korea from 2008 to 2016. High incidence regions are in Gyeongbuk, Gyeongnam, and Gangwon provinces. In contrast, many low incidence regions are located in Seoul metropolitan area.

Table 1 shows descriptive statistics and references for the variables used in the analysis. Annual TB incidence rate for Si, Gun, and Gu (city and county-level administrative areal units) is used for the dependent variable. The annual incidence rate is calculated by dividing new TB cases with the population size for each areal unit. The equation is as follows,

$$Incidence\;Rate=\frac{Total\;number\;of\;new\;disease\;cases\;during\;a\;given\;period\;}{The\;total\;population\;(at\;risk)\;during\;the\;same\;period\;}\times 10^{5}\;(population\;unit)$$

**Table 1:** Descriptive statistics and references for the variables.

Category		Variables	Unit	Mean (9 years)	References	Source (homepage)
Dependent variable		TB Incidence Rate	Cases/Population (100,000)	81.34		KCDC, http://www.cdc.go.kr
Independent Variables	Social Factors	Population composition ratio	$\frac{Female\;pop\;(20-39)}{Pop\;above\;65}$	1.06	[11,32–35]	KOSIS, http://www.kosis.kr
		Population growth rate	%	0.43	[36–39]	KOSIS, http://www.kosis.kr
		Health insurance payment	1M KRW^a^	27,209	[3–5]	NHIS, http://nhiss.nhis.or.kr
		The number of people per medical personnel	-	11.12	[17,40,41]	NHIS, http://nhiss.nhis.or.kr
	Environmental Factors	Sulfur dioxide	Ppm	0.005	[27,42–44]	NIER, http://www.nier.go.kr
		Jan. to April mean temperature	°C	2.25	[45–47]	NIER, http://www.nier.go.kr

^a^1M KRW: One million Korean won.

The independent variables included in this study represent social and environmental factors including demographic and socioeconomic characteristics, access to public health services, air quality, and climatic averages.

Population composition ratio represents regional demographic trends by comparing reproduction potential with elderly population size [32]. The ratio is calculated by dividing the female population aged 20–39 by the total population aged 65 and over. A ratio of less than 0.5 is considered a regional population crisis because the number of births would be less than the size of the elderly population, leading to regional extinction [32].

Individuals above the age of 65 are considered more susceptible to respiratory diseases such as TB [11,33]. The percent of the population aged 65 and over in South Korea has increased from 10.2% in 2008 to 13.5% in 2016, and is expected to reach 33.9% in 2040 [34]. This trend suggests that South Korea’s population is aging rapidly. The variable presents the aging characteristics of Korean demography. In contrast to the population above 65, the female population aged 20–39 decreased dramatically from 34.7% in 2000 to 26.7% in 2015 [35]. Both data were collected and calculated from the Korean Statistical Information Service (KOSIS, http://www.kosis.kr).

Literature about the relationship between population growth rate and TB provides two opposite perspectives: 1) In regions with high population growth, higher TB incidence risks are expected. Given TB’s contagious infection feature, an increased probability of people’s physical contact leads to higher TB infection rates [36]. On the other hand, 2) urban areas, experiencing high population growth, tend to have better sanitation and healthcare infrastructures compared to rural areas [48]. Consequently, urban areas have a lower TB incidence rate. The population growth rate variable offers an promising alternative to these two opposing perspectives for correlating demographic trends with TB incidence.

In Korea, the population growth rate is mostly decided by people’s migration and corresponding inbound and outbound population. Natural population increase is considered negligible in population growth rate [37,38]. For reference, Korea’s total fertility rate was 0.92 in 2019, the lowest in the world [39]. The data was acquired from the Korean Statistical Information Service (KOSIS, http://www.kosis.kr).

The health insurance variable is used as a proxy to reflect the economic status of the population for each area. Many studies show higher TB incidence risks in population with low economic status [3–5]. Korea has a national health insurance system, collecting contributions at city and county level. All citizens make insurance payments based on their income: the higher they make, the more they pay. Naturally, well off neighborhoods pay relatively more expensive health insurance costs. The data were obtained from the National Health Insurance Sharing Service (NHIS, https://nhiss.nhis.or.kr).

The number of people per medical personnel measures the level of health care and accessibility to medical services. Obtained from the NHIS, the medical personnel data includes doctors, nurses, and pharmacists. TB treatment requires regular check-ups and continuous drug treatment. Inappropriate treatment may cause secondary infection and recurrence [41].

As an environmental factor, sulfur dioxide is a proxy for air pollution. As a leading cause of respiratory disease, sulfur dioxide causes pulmonary diseases such as bronchitis, pulmonary edema, and pneumonia [42]. Several studies indicated its effects on TB incidence in Korea [27,43,44]. The sulfur dioxide data were collected from 227 air monitoring stations built and managed by the National Institute of Environmental Research (NIER, https://www.nier.go.kr).

Temperature is closely related to respiratory diseases in two ways. High temperatures exacerbate chronic respiratory illness. Low temperatures weaken immune system leading to higher respiratory disease and death rate [45,46]. Tracking TB incidences in China from 2005 to 2013, most new TB incidences occurred in the 1st quarter (Jan. to April) of the year because of relatively weak immune system [47]. This study extracted 1st quarter average temperature data from the metrological stations of 59 branches of the Atmospheric Environment Annual Report of NIER.

### Spatial autocorrelation

Spatial autocorrelation is a measure of the extent to which adjacent spatial units are more similar to one another than they are to remote spatial units [22]. Adjacent regions influence each other, as TB incidence of one region affects the incidence of the neighbors [49]. Spatial autocorrelation is observed in spatial patterns of TB incidence in Korea [44]. Detecting spatial autocorrelation of diseases helps to identify epidemic characteristics and influences between disease and socio-environmental factors [49]. Several studies in Korea tried to find and represent spatial characteristics of disease incidences by accounting for the spatial autocorrelation [30,44,50]. This study applied Moran’s I to measure spatial autocorrelation of TB incidence [22,51].

### Eigenvector spatial filtering

Eigenvector spatial filtering is a spatial regression model that explains the spatial autocorrelation of residuals by adding selected eigenvectors to the model [52]. Eigenvectors used in this study are generated by the Simultaneous Autoregressive (SAR) process [29,52]. SAR model based eigenvector spatial filtering leads to non-biased parameter estimation, presuming eigenvectors are mutually independent [29]. SAR process and corresponding eigenvector spatial filtering are as follows [29],

**Table:** 

SAR process (*I*−*ρV*)*y* = (*I*−*ρV*)*Xβ*+*ε* *y* = *Xβ*+(*I*−*ρV*)^−1^*ε* $y=X\beta +\underset{\mathrm{misspecified}\;\mathrm{term}}{\underset{︸}{\sum_{k=1}^{\infty }\rho^{k}V^{k}\varepsilon }}+\varepsilon$	Eigenvector spatial filtering $y=X\beta +\underset{\begin{array}{c} \mathrm{Account}\;\mathrm{for}\\ \mathrm{misspecified}\;\mathrm{term} \end{array}}{\underset{︸}{E_{SAR}\gamma }}+\varepsilon$

The misspecified term, $\sum_{k=1}^{\infty }\rho^{k}V^{k}\varepsilon$, in SAR process is accounted for by E_SAR_*γ* in eigenvector spatial filtering. The misspecified term is a set of unspecified and/or missing exogenous variables, jointly representing a spatial pattern in relation to the spatial weight matrix *V* [29]. In the equation above, *X* is a matrix of independent variables, and *β* is a vector of estimated parameters. Selected eigenvectors, E_SAR_, function as proxy variables accounting for spatial effect in the data [29]. *ρ* and *γ* are spatial autocorrelation parameter in SAR process and eigenvector spatial filtering process, respectively. *V* is a spatial weights matrix, and ε is a random white noise without spatial autocorrelation. The spatial weights matrix *V* is defined by the queen contiguity: 1 if neighbors had a common edge or vertex, and 0 otherwise [53].

### Hotspot and coldspot analysis

Getis-Ord $G_{i}^{*}$ is applied to detect hotspots and coldspots of TB incidence. Getis-Ord $G_{i}^{*}\;$ is defined as follows,

$$G_{i}^{*}=\frac{\sum_{j=1}v_{ij}x_{j}-\bar{x}\sum_{j=1}v_{ij}}{S\sqrt{\frac{\left[n\sum_{j=1}v_{ij}^{2}-(\sum_{j=1}v_{ij}{)}^{2}\right]}{n-1}}}$$

*n* is the number of spatial units, $\bar{x}$ is the average value of all *x*_*j*_, and S is the standard deviation of *x*_*j*_. *v*_*ij*_ is a value of spatial weight matrix. $G_{i}^{*}$ compares the sum of *x*_*i*_’s neighboring values with the overall mean $\bar{x}$ [31,54]. Positive and statistically significant $G_{i}^{*}s$ are hotspots. On the contrary, statistically significant negative $G_{i}^{*}s$ are coldspots. Moran’s *I* may also detect local spatial clusters. Compared to Getis-Ord $G_{i}^{*}$, however, Moran’s *I* is more appropriate to find local spatial outliers as it compares only neighboring values rather than the overall mean.

This study used the statistical software program R 3.4.2 for statistical analysis and the ESRI ArcGIS pro for map generation and visualization. The *ME(Moran Eigenvector GLM Filtering)* function in R package *spdep* was used to generate eigenvectors.

## Results and discussion

### Eigenvector spatial filtering model (ESFM) results

**Fig 2:**
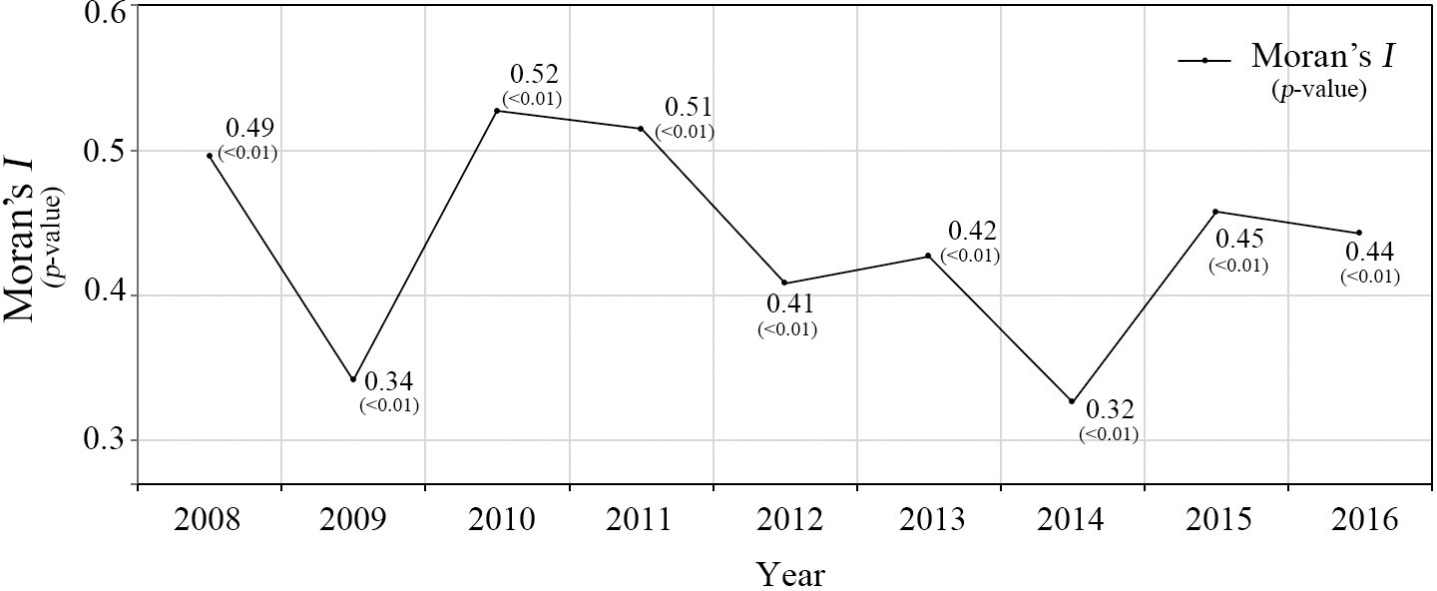
Spatial autocorrelation of TB incidence rate (2008–2016).

Fig 2 presents the changes in spatial autocorrelation of TB incidence rate from 2008 to 2016 using Global Moran’s *I*. Throughout the years, Moran’s *I* appears statistically significant (*p*-value < 0.01), representing strong positive spatial autocorrelation of TB incidence in the study area.

Table 2 presents the result of the eigenvector spatial filtering model (ESFM) in comparison with OLS model. Seeing *R*^2^ and the AIC (Akaike Information Criterion) values, ESFM presents improved model fit compared to OLS model throughout the years. Moran’s *I* of residuals presents that spatial autocorrelation in OLS residuals is effectively removed in ESFM, making residuals i.i.d. (Independently and Identically Distributed).

**Table 2:** Estimation results of the eigenvector spatial filtering model.

Variables		2008	2009	2010	2011	2012	2013	2014	2015	2016
Population composition ratio		-5.02*	-7.25**	-2.89	-4.28	-7.30*	-6.17*	-7.42	-1.01*	-7.66**
Population growth rate		-1.30*	-2.94**	-2.21**	-3.18**	-2.26*	-2.74**	-2.18**	-1.97**	-1.55**
Health insurance payment		-13.58**	-19.02**	-15.67**	-15.54**	-15.01**	-15.05**	-23.92*	-17.70*	-19.04**
The number of people per medical personnel		1.01**	1.01*	0.85**	0.44**	0.49**	0.36*	0.83**	0.05	0.08
Sulfur dioxide		14.73	27.88*	26.25**	29.77**	15.16	28.48**	5.87	9.81*	3.45
Jan. to April mean temperature		2.44**	2.92*	3.26**	3.25**	2.30*	0.87	0.13	0.36	0.23
**Model Fit**										
*R*^2^	ESFM (OLS)	0.44 (0.31)	0.42 (0.31)	0.50 (0.35)	0.51 (0.38)	0.40 (0.30)	0.41 (0.31)	0.35 (0.33)	0.55 (0.50)	0.52 (0.50)
AIC	ESFM (OLS)	2195.31 (2224.92)	2407.16 (2431.86)	2202.84 (2247.02)	2221.40 (2263.64)	2278.56 (2305.83)	2239.66 (2268.54)	2405.42 (2407.92)	2191.00 (2218.65)	2234.07 (2240.13)
Moran’s *I* of Residuals	ESFM (OLS)	-0.06 (0.14**)	-0.02 (0.11**)	-0.04 (0.17**)	-0.04 (0.16**)	-0.02 (0.10**)	-0.05 (0.11**)	-0.04 (0.12**)	-0.02 (0.12**)	-0.02 (0.11**)

*p-value < 0.05

**p-value<0.01; AIC: Akaike information criterion; OLS: Ordinary least squares regression; ESFM: Eigenvector spatial filtering model.

Minimizing Moran’s *I* method is applied in eigenvector selection in this study [29]. This method has the advantage of leading a robust and smaller number of eigenvectors [29]. Given that the selection aims to remove spatial autocorrelation in residuals, the procedure of adding eigenvector selection is finished when Moran’s *I* value decreases to zero [29].

**Fig 3:**
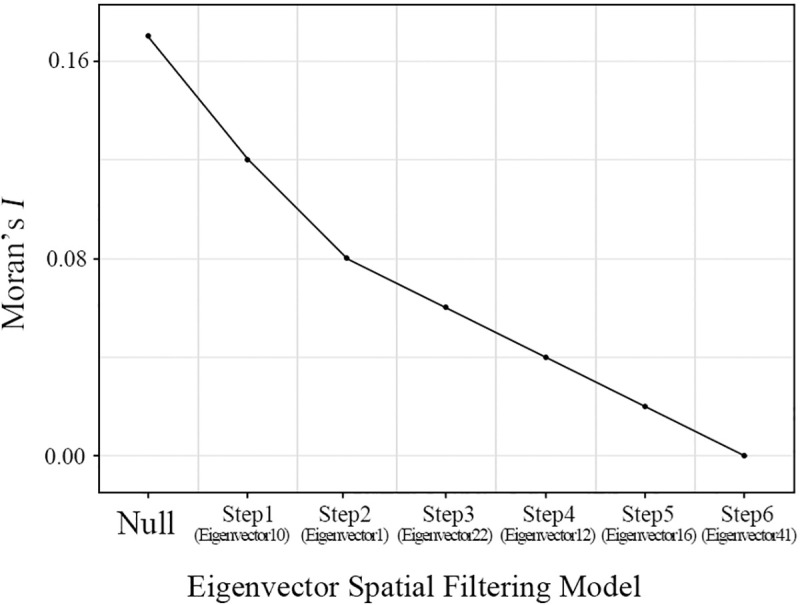
Spatial autocorrelation in ESFM (2010).

Fig 3 presents the spatial autocorrelation as eigenvectors added in the model for the year 2010. X-axis shows eigenvector selection steps, and Y-axis shows corresponding Moran’s *I* values for each step. A total 6 eigenvectors are selected (eigenvector no. 10, 1, 22, 12, 16, and 41).

### ESFM results interpretation

The population growth rate and health insurance payment show consistent statistical significance during the entire study period. The rest of the variables show changes in statistical significance over the periods.

The population composition ratio represents an evident relationship between age & sex specified demography and TB incidence. The ratio keeps showing statistically significant coefficients with minor exceptions in 2010, 2011, and 2014. The negative coefficients reflect strong and statistically significant correlation (0.71, *p*-value < 0.01) between population over 65 ratio and TB incidence. In addition, regions where the population 65 years and over outnumbers females aged 20–39 show higher TB incidence rates. In these regions, natural population declines accelerate, decreasing the quality of social infrastructure and the availability of medical services [55]. Consequently, people living in regions where the population 65 years and over outnumbers females aged 20–39 do not have sufficient qualities of medical services, leading to an increase in TB incidence [56].

Given demographic characteristics in Korea, controlling TB incidence is challenging work because of the rapid aging trends. Population over 65 ratio was 14.9% in 2019 and is expected to reach 33.9% in 2040 [34]. Besides, the female population aged 20–39 declined dramatically from 2010 to 2015 [35], leading to the lowest birth rate in history [39]. The low birth rate accelerates the aging population trend in Korea. As a result, the ratio of a vulnerable population to TB incidents is radically increasing year by year.

The population growth rate shows a negative relationship with the TB incidence throughout the study period. For example, in 2016, the regression coefficients of the population growth rate indicates that TB incidence rate decrease -1.55 according to one percent increase of the population growth rate. Regions with a high population growth rate show a low TB incidence rate. This implies that these regions have good healthcare services, facilities, and proper sanitation. Population migration toward cities with better infrastructure leads to regional population growth. In particular, Seoul metropolitan area and Sejong are ranked 3^rd^ and 1^st^ in population growth rate with significantly low TB incident rates [57].

The regression coefficient of health insurance payment shows that TB incidence rate decrease from -23.92 to -13.58 during the entire period according to the increase one million Korean won of health insurance payment [S1 Fig]. It confirms that regions in lower economic status are more vulnerable to TB incidence. People in these regions continue to suffer not only a high risk TB infection, but also high recurrence rate from relatively poor healthcare services [58,59]. This incidence pattern has been referred to as a “vicious cycle” [58]. Not having sufficient healthcare services and corresponding high TB incidence risks, many regions are in the vicious cycle of TB in the study area [60]. Consequently, prolonged TB treatment and MDR (Multidrug-resistant) TB incidence risks are higher in these regions [61]. TB incidence driven by economic status and corresponding vicious cycle in the regions would widen TB incidence gap as the result of regional economic polarization in Korea.

The number of population per medical personnel shows that lower medical capacity is linked to more TB incidence in Korea. Aa a representative region, Gyeongbuk province shows the lowest medical capacity (2.1 doctors per 1,000 people) with the highest TB incidence rate (a rate of 115.44 per 100,000 persons) [62]. In contrast, Seoul has twice the medical capacity (4.4 doctors per 1,000 people) compared to Gyeongbuk province with a lower TB incidence rate (a rate of 74.06 per 100,000 persons) [62].

As environmental variables, sulfur dioxide level and Jan. to April temperature do not show a consistent relationship with TB incidence. Although statistically inconsistent, a high sulfur dioxide level increases TB incidence risk. Contrary to sulfur dioxide, Jan. to April temperature does not provide considerable epidemiological value.

### Spatial pattern and trend analysis

**Fig 4:**
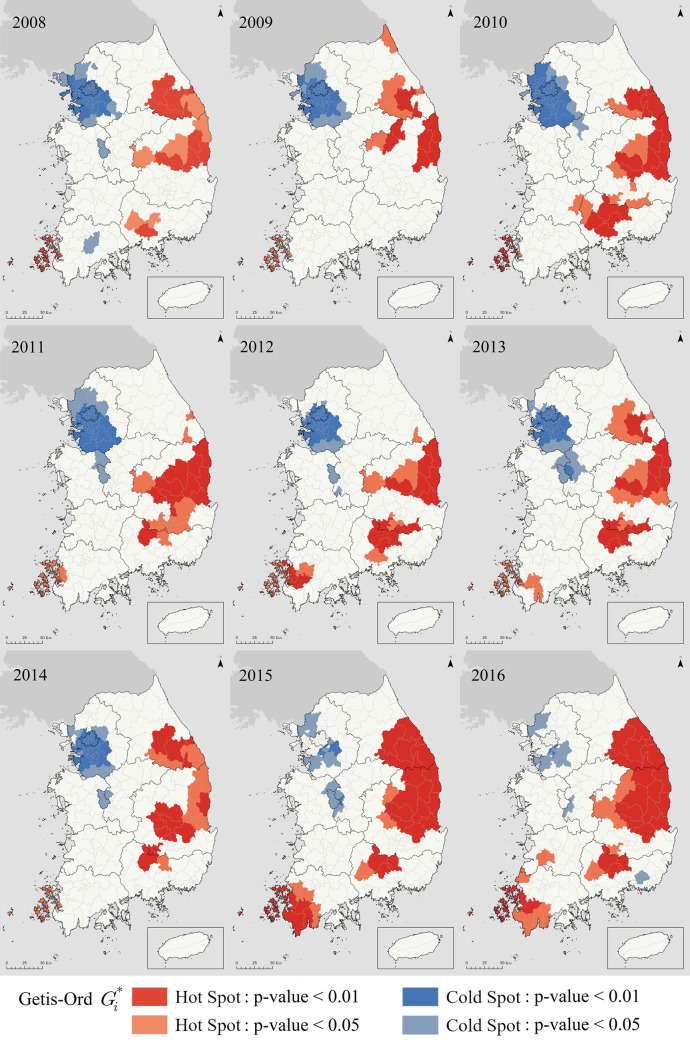
Spatial patterns of TB hotspot and coldspot (2008–2016).

Fig 4 shows the spatial patterns of TB hotspots and coldspots measured by $G_{i}^{*}$ statistics. Forming clusters, hotspots are mostly located in the eastern part of Korea. These areas are characterized by large outbound populations and low socio-economic conditions [57,63]. In contrast, most coldspots are located in Seoul metropolitan area and Sejong, which have the best urban infrastructure and large population growth with affluent socio-economic status. Seoul metropolitan area shows the largest population growth and hospital rooms [57,64]. Sejong has shown the highest inbound population aged 30s and birthrate since 2012 [65]. It is notable that Sejong is a master-planned city founded on a barren wasteland in 2012, implemented by the former president Roh. Relocating major government administrative functions from Seoul, the establishment triggered the population movements and economic developments [66,67].

Korea’s TB clustering pattern represents the specific social determinants of TB incidence in this country [68]. Regional variation in demographic and socioeconomic variables correlate to spatial variation in TB incidence. The causes of these differences are as follows. First, people’s migration has widened the gap of aged related regional demography [38], exacerbating TB incidence in certain areas. Given ageing as a risk factor for TB incidence [69], regions with higher rate of aged population are TB hotspots with continuing outbound population. Second, regional differences in socio-economic status affected medical capacities and corresponding medical services, enlarging regional gaps in TB incidence. It is well-known that socio-economic conditions are one of the most important determinants in TB incidence [68].

**Fig 5:**
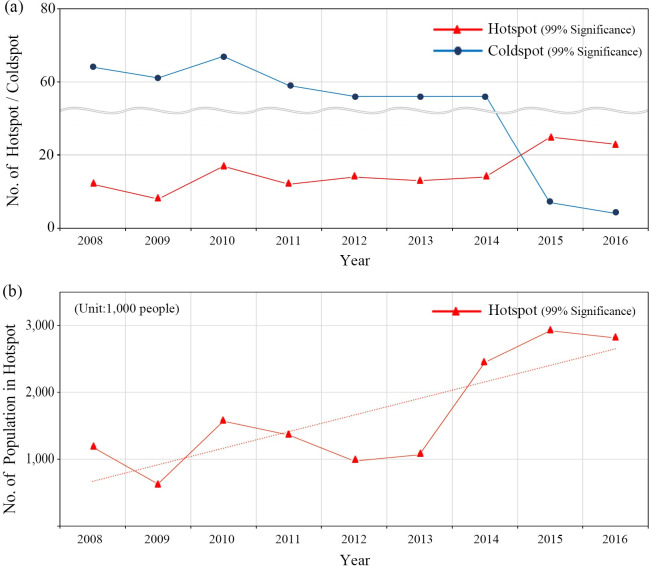
(a) No. of hotspot and coldspot; (b) No. of population in hotspot.

Fig 5 indicates (a) changes in the numbers of hotspots and coldspots (99% significance) and (b) corresponding population size at risk in hotspots. The number of hotspots increased about 90% throughout the study period, and the corresponding population size increased about 2.7 times. The growing number of hotspots implies that the number of high-risk TB regions and the potential TB transmission risk have increases. This suggests that consistent and extended health and medical cares are required in the areas.

## Conclusions

This study investigated TB incidence with socio-environmental factors using eigenvector spatial filtering model. Space-time patterns of TB incidence were explored using Getis-Ord $G_{i}^{*}$ statistics. The result presented that the influence of socio-economic variables (population composition ratio, population growth rate, health insurance payment, and the number of people per medical personnel) were more robust than environmental variables (sulfur dioxide and Jan. to April mean temperature). In particular, demographic and socio-economic conditions are the main factors in making spatial differences of TB incidence. Both increasing population over 65 and decreasing female population aged 20–39 are a potential threat to TB control in the future. Environmental factors related to TB showed minor significance. However, it requires further in-depth studies.

This study has limitations, using aggregated incidence data by administrative regions. Using areal data can not provide an exact pinpointing location of hotspots and corresponding local human and natural conditions. Furthermore, lack of specific patients data, such as sex, age, occupation, and health condition, constrains in-depth explanations about Korea’s epidemiological characteristics of TB.

## Supporting information

S1 Fig Plot of eigenvector spatial regression coefficients with 95% confidence intervals. (TIF)
