# Supplementary figures and images for: Spatial pattern of tuberculosis (TB) and related socio-environmental factors in South Korea, 2008-2016
Source: PLoS One. 2021 Aug 5;16(8):e0255727. doi: 10.1371/journal.pone.0255727 (PMC8341643; doi:10.1371/journal.pone.0255727)

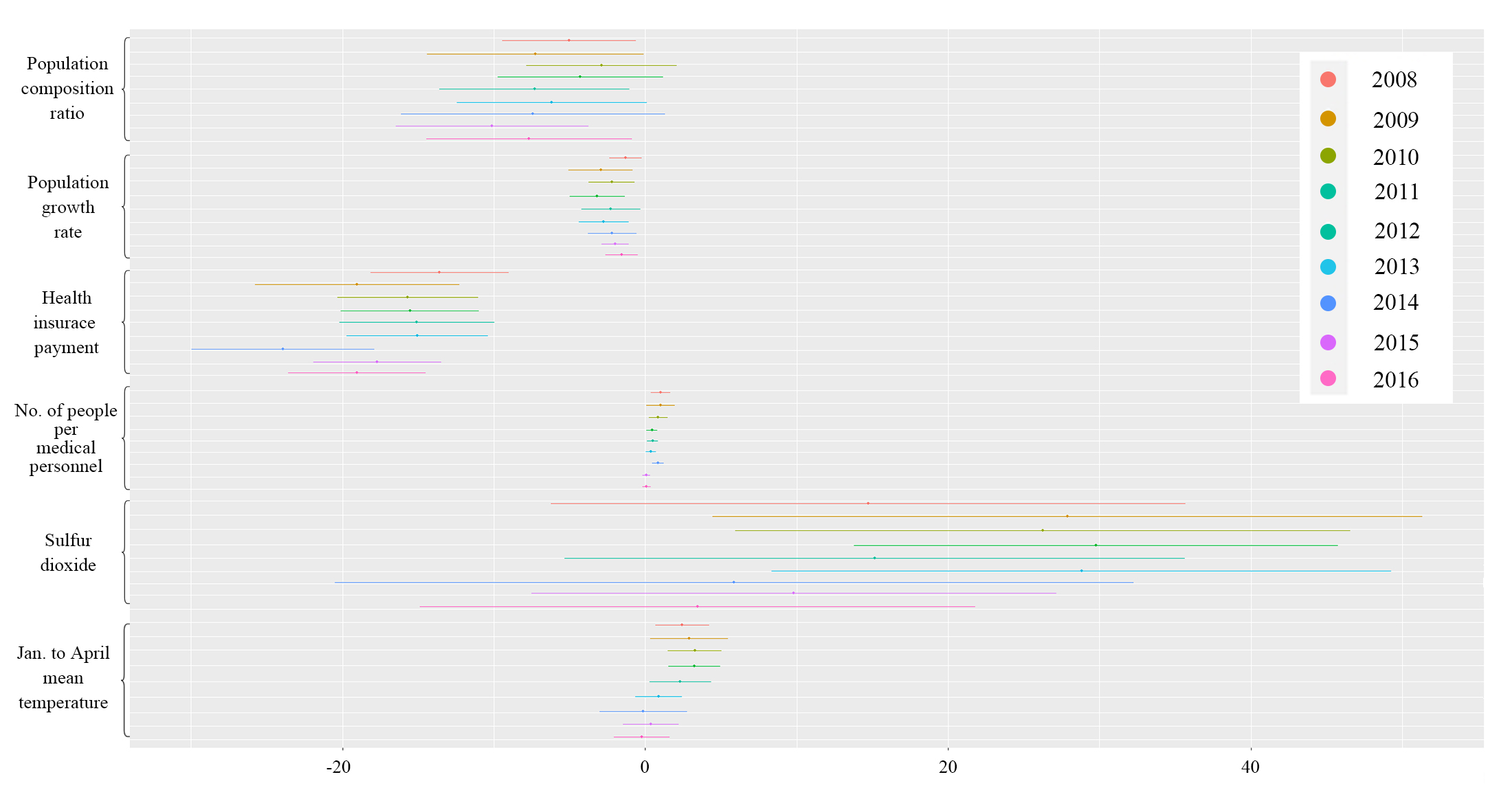

Supplement: S1 Fig — (TIF) [file pone.0255727.s001.tif]
